# Prognostic value of preoperative hematologic biomarkers in urothelial carcinoma of the bladder treated with radical cystectomy: a systematic review and meta-analysis

**DOI:** 10.1007/s10147-020-01690-1

**Published:** 2020-05-26

**Authors:** Keiichiro Mori, Noriyoshi Miura, Hadi Mostafaei, Fahad Quhal, Reza Sari Motlagh, Ivan Lysenko, Shoji Kimura, Shin Egawa, Pierre I. Karakiewicz, Shahrokh F. Shariat

**Affiliations:** 1 Department of Urology, Medical University of Vienna, Währinger Gürtel 18-20, 1090 Vienna, Austria; 2 Department of Urology, The Jikei University School of Medicine, Tokyo, Japan; 3 Department of Urology, Ehime University Graduate School of Medicine, Ehime, Japan; 4 Research Center for Evidence Based Medicine, Tabriz University of Medical Sciences, Tabriz, Iran; 5 Department of Urology, King Fahad Specialist Hospital, Dammam, Saudi Arabia; 6 Cancer Prognostics and Health Outcomes Unit, University of Montreal Health Centre, Montreal, Canada; 7 Institute for Urology and Reproductive Health, I.M. Sechenov First Moscow State Medical University, Moscow, Russia; 8 Department of Urology, Weill Cornell Medical College, New York, NY USA; 9 Department of Urology, University of Texas Southwestern, Dallas, TX USA; 10 Karl Landsteiner Institute of Urology and Andrology, Vienna, Austria; 11 Department of Urology, Second Faculty of Medicine, Charles University, Prague, Czech Republic; 12 Department of Urology, University of Jordan, Amman, Jordan; 13 European Association of Urology Research Foundation, Arnhem, The Netherlands

**Keywords:** Urothelial carcinoma of the bladder, Hematologic biomarker, Meta-analysis

## Abstract

This systematic review and meta-analysis aimed to assess the prognostic value of preoperative hematologic biomarkers in patients with urothelial carcinoma of the bladder treated with radical cystectomy. PUBMED, Web of Science, Cochrane Library, and Scopus databases were searched in September 2019 according to the Preferred Reporting Items for Systematic Review and Meta-analysis statement. Studies were deemed eligible if they compared cancer-specific survival in patients with urothelial carcinoma of the bladder with and without pretreatment laboratoryabnormalities. Formal meta-analyses were performed for this outcome. The systematic review identified 36 studies with 23,632 patients, of these, 32 studies with 22,224 patients were eligible for the meta-analysis. Several preoperative hematologic biomarkers were significantly associated with cancer-specific survival as follows: neutrophil − lymphocyte ratio (pooled hazard ratio [HR]: 1.20, 95% confidence interval [CI]: 1.11–1.29), hemoglobin (pooled HR: 0.87, 95% CI 0.82–0.94), C-reactive protein (pooled HR: 1.44, 95% CI 1.26–1.66), De Ritis ratio (pooled HR: 2.18, 95% CI 1.37–3.48), white blood cell count (pooled HR: 1.05, 95% CI 1.02–1.07), and albumin-globulin ratio (pooled HR: 0.26, 95% CI 0.14–0.48). Several pretreatment laboratory abnormalities in patients with urothelial carcinoma of the bladder were associated with cancer-specific mortality. Therefore, it might be useful to incorporate such hematologic biomarkers into prognostic tools for urothelial carcinoma of the bladder. However, given the study limitations including heterogeneity and retrospective nature of the primary data, the conclusions should be interpreted with caution.

## Introduction

Urothelial carcinoma of the bladder (UCB) is the ninth most commonly diagnosed cancer worldwide [1]. Radical cystectomy (RC) with lymph node dissection is the mainstay treatment for very high-risk non-muscle-invasive and muscle-invasive UCB [2, 3]. Despite definitive therapy with curative intent, the 5-year overall survival of patients remains below 60% [4, 5]. Thus, various clinical and pathologic factors have been identified to assist in the risk stratification of UCB patients, thereby facilitating clinical decision-making regarding treatment intensification, follow-up and patient counselling [6, 7]. Currently, the majority of these factors are pathological features such as tumor stage, grade, lymph node status, concomitant carcinoma in situ, variant histology, surgical margin status, and lymphovascular invasion. Unfortunately, the accuracy of outcome prediction with these factors remains suboptimal, probably due to their failure to capture the full biologic potential of host-tumor interactions [8]. In addition, clinical, radiologic, and pre-RC pathologic factors have significant limitations, and do not allow for optimal clinical decision making [6, 9]. Therefore, there remains a need to identify other potential prognostic markers, in particular preoperatively, to improve the stratification of patients with muscle-invasive UCB.

Recently, there has been a surge of interest in the prognostic role of hematologic biomarkers in patients undergoing RC. Current research has suggested that hematologic biomarkers, such as neutrophil–lymphocyte ratio (NLR), C-reactive protein (CRP), lymphocyte-monocyte ratio (LMR), platelet-lymphocyte ratio (PLR), and hemoglobin (Hb), may have prognostic value in patients with UCB [3, 10]. However, the prognostic significance of hematologic biomarkers remains to be established in UCB treated with RC. Therefore, this systematic review and meta-analysis were conducted to summarize the available evidence as well as to determine whether preoperative hematologic biomarkers may help predict oncological outcomes in patients with UCB treated with RC. If such biomarkers are predictive of outcomes in this patient population, a panel of these markers could help identify and classify patients, as well as aid in the selection of patients for novel therapies that rely heavily on host-tumor interaction.

## Methods

### Search strategy

The systematic review and meta-analysis were performed according to the Preferred Reporting Items for Systematic Reviews and Meta-analyses (PRISMA) statement [11]. The PubMed, Web of Science, Cochrane Library, and Scopus databases were searched in September 2019 to identify reports on the prognostic value of blood-based biomarkers in UCB. The keywords used in our search strategy were: (cystectomy) AND (multivariate OR multivariable) AND (survival OR mortality): The primary outcome of interest was cancer-specific survival (CSS). Initial screening was performed independently by two investigators based on the titles and abstracts to identify ineligible reports, and reasons for exclusions were noted. Potentially relevant reports were subjected to a full-text review and the relevance of the reports was also confirmed after the data extraction process. Disagreements were resolved via consensus with the additional investigator.

### Inclusion and exclusion criteria

Studies were included if they investigated patients treated for UCB with preoperative laboratory abnormalities (Patients) who had received​ radical cystectomy (Intervention) compared to those without preoperative laboratory abnormalities (Comparison) to assess the independent predictive value of blood-based biomarkers on CSS (Outcome) utilizing multivariate Cox regression analysis (Study design) in nonrandomized observational, randomized, or cohort studies. We excluded reviews, letters, editorials, meeting abstracts, replies from authors, case reports and articles not published in English. In cases of duplicate publications, the higher quality or the most recent publication was selected. References of included manuscripts were further scanned for additional studies of interest.

### Data extraction

Two investigators independently extracted the following information from the included articles: first author’s name, publication year, recruitment country, period of patient recruitment, number of patients, age, sex, study design, disease stage, oncological outcome, follow-up duration, pathological T stage, adjuvant chemotherapy, neoadjuvant chemotherapy, conclusion, and type of biomarkers. Subsequently, the hazard ratios (HR) and 95% confidence intervals (CI) of blood-based biomarkers associated with each of the outcomes were retrieved. The HRs were extracted from the multivariate analyses and all discrepancies regarding data extraction were resolved by consensus with the additional investigator.

### Quality assessment

The Newcastle–Ottawa Scale (NOS) was used to assess the quality of the included studies in accordance with the Cochrane Handbook for systematic reviews of interventions for included non-randomized studies [12, 13]. The scale rates following three factors: Selection (1–4 points), Comparability (1–2 points) and Exposure (1–3 points), with total scores ranging from 0 (lowest) to 9 (highest). The main confounders were identified as the important prognostic factors of CSS. The presence of confounders was determined by consensus and　review of the literature. Studies with scores of more than 6 were identified as “high-quality” choices.

### Statistical analyses

Forest plots were used to assess the multivariate HRs and summarize them to describe the relationships between blood-based biomarkers and CSS. Studies were not considered in the meta-analysis if they used Kaplan–Meier log-rank, univariate Cox proportional hazard regression, or general logistic regression analyses. In studies with only HRs and P-values, we calculated the corresponding 95% CIs [14, 15]. Heterogeneity among the outcomes of included studies in this meta-analysis was evaluated by using Cochrane’s *Q* test and the *I*^2^ statistic. Significant heterogeneity was indicated by a *P* < 0.05 in Cochrane’s *Q* tests and a ratio > 50% in *I*^2^ statistics. We used fixed-effects models for the calculation of pooled HRs for non-heterogeneous results [16–18]. Publication bias was assessed using funnel plots. All statistical analyses were performed using Stata/MP 14.2 (Stata Corp., College Station, TX); statistical significance level was set at *P* < 0.05.

## Results

### Study selection and characteristics

**Fig. 1:**
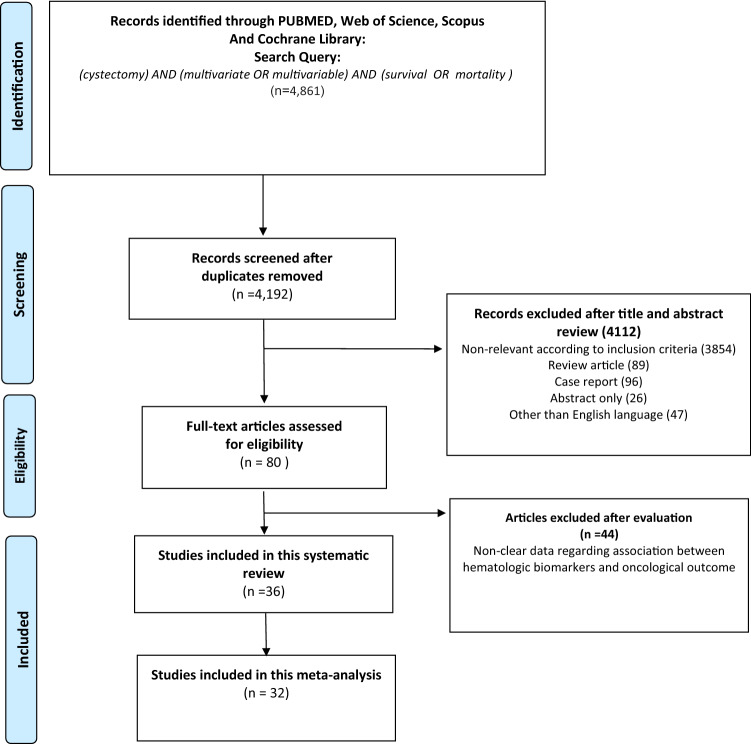
The Preferred Reporting Items for Systematic Reviews and Meta-analyses (PRISMA) flow chart, detailing the article selection process

Our initial search identified 4861 records, and after removing of duplicates, 4192 remained (Fig. 1). A total of 4112 articles were excluded after screening the titles and abstracts, and a full-text review was performed for 80 articles. After applying the selection criteria, we identified 36 articles with 23,632 patients for the systematic review, of which, 32 articles with 22,224 patients were used for the meta-analysis [10, 19–53]. The extracted data from the 36 studies are outlined in Tables 1 and 2. All included studies had a retrospective design and were published between 2002 and 2019, with 13 studies being from Europe, 5 from North America, 15 from Asia and 3 from international collaboration. The median age and follow-up ranged from 60.7 to 72 years, and 14 to 132 months, respectively; 19,185 of the studied patients were male and 4447 were female. The studies had a median NOS score of 7 (6–7)0.2329.

**Table 1:** Study characteristics

Author	Year	Region	Period	N	D	Type of markers evaluated (cut off values)	Significant markers	NOS
Buisan	2016	Spain	2007–2015	75	R	NLR (continuous)	NLR	7
Calvete	2019	Spain	2000–2015	121	R	Hb (13 g/dL)	Hb	7
Chipollini	2016	USA	2008–2015	1026	R	Hb (NR)	Hb	7
D'Andrea	2017	International	1990–2012	4198	R	LMR (3.5), NLR (2.7)	LMR, NLR	7
Ergani	2015	Turkey	2009–2014	148	R	Hb (12.2 g/dL)	Hb	6
Gershman	2016	USA	1980–2008	2086	R	Hb (continuous)	Hb	7
Gierth	2015	Germany	2001–2011	684	R	Hb (male 13 g/dL, female 12 g/dL)	Hb	7
Gondo	2012	Japan	2000–2009	189	R	Hb (11.5 g/dL), NLR (2.5), Plt (300,000/uL), LDH (360u/L), CRP (0.5 mg/dL), Neu (6500/uL), Lym (1500/uL)	Hb, NLR	7
Gorgel	2017	Turkey	2006–2016	153	R	De Ritis (1.3)	De Ritis	6
Grimm	2015	Germany	2004–2013	664	R	CRP (0.5 mg/dl), Hb (13.4 g/dl)	CRP, Hb	7
Ha	2019	Korea	2008–2013	118	R	De Ritis (1.3)	De Ritis	7
Hermanns	2014	Canada	1992–2012	424	R	Hb (continuous), NLR (3), Plt (continuous)	Hb, NLR, Plt	7
Jo	2016	Korea	2003–2014	200	R	Hb (male 13 g/dL, female 12 g/dL)	Hb	7
Jokisch	2019	Germany	2004–2017	866	R	Plt (400,000/uL)	Plt	7
Kang	2017	Korea	1999–2012	385	R	NLR (2.5)	NLR	6
Kluth	2015	International	1979–2012	967	R	Alb (continuous), Hb (continuous), LDH (continuous), Plt (continuous), WBC (continuous)	Alb, Hb, LDH, Plt, WBC	6
Ku	2015	Korea	1999–2011	419	R	Alb (3.5 g/dL), Lym (1000/uL), Plt (400,000/uL), CRP (10 mg/dL), WBC (11,000/uL), Neu (7500/uL)	Alb, Lym, Plt	7
Kwon	2014	Korea	1990–2012	714	R	Alb (3.5 g/dL)	Alb	7
Lambert	2013	USA	2004–2011	187	R	Alb (3.5 g/dL)	Alb	7
Liu J	2016	China	2000–2013	296	R	AGR (1.6), Alb (continuous), Hb (continuous), Neu (continuous), Plt (continuous), WBC (continuous)	AGR, Alb, Hb, Neu, Plt, WBC	7
Liu Z	2017	China	2009–2013	189	R	AGR (1.55)	AGR	7
Lucca	2016	International	1979–2012	4061	R	NLR (2.7)	NLR	7
Matsumoto	2017	Japan	1990–2013	594	R	eGFR (60 mL/min/1.73m^2^)	eGFR	7
Miyake	2017	Japan	2006–2016	117	R	NLR (2.6), PLR (150), MLR (0.3)	NLR, PLR	6
Moschini	2014	Italy	1995–2012	906	R	Hb (12 g/dL), Leukocyte (1000/uL), Plt (400,000/uL)	Hb, Leukocyte, Plt	7
Ozcan	2015	Turkey	1990–2013	286	R	Leukocyto (11,000/uL), NLR (2.5), Neu (7700/uL), Lym (1500/uL)	Leukocyto, NLR, Neu, Lym	7
Rajwa	2018	Poland	2003–2015	144	R	LMR (continuous), NLR (continuous), PLR (continuous)	LMR, NLR, PLR	6
Schubert	2015	Germany	1999–2009	246	R	Hb (12 g/dL)	Hb	7
Sejima	2013	Japan	2003–2011	249	R	Alb (continuous), CRP (continuous), Hb (continuous), LDH (continuous)	Alb, CRP, Hb	7
Tan	2017	Singapore	2002–2012	84	R	NLR (2.7), Hb (male13.5 g/dL, female 12.5 g/dL)	NLR	7
Todenhofer	2012	Germany	1999–2010	258	R	PLT (450,000/uL), Hb (male14g/dL, female 12 g/dL)	PLT	7
Un	2018	Turkey	2002–2012	296	R	Hb (NR), NLR (2.7)	Hb, NLR	7
Viers	2014	USA	1994–2005	899	R	NLR (continuous)	NLR	7
Yang	2002	China	1987–1997	310	R	Alb (3 g/dL), ALP (100U/L), Cr (1.5 mg/dL), Hb (10 g/dL), Plt (100,000/uL), WBC (10,000/uL)	Alb, ALP, Cr, Hb, Plt, WBC	7
Yoshida	2016	Japan	1995–2013	302	R	LMR (NR)	LMR	7
Yuk	2019	Korea	1991–2015	771	R	De Ritis (1.1)	De Ritis	7

*AGR* albumin-globulin ratio, *Alb* albumin, *ALP* alkaline phosphatase, *Cr* creatinine, *CRP* C-reactive protein, *D* design, *eGFR* estimate glomerular filtration rate, *Hb* hemoglobin, *LDH* lactate dehydrogenase, *LMR* lymphocyte-to-monocyte ratio, *Lym* lymphocyte, *MLR* monocyte-lymphocyte ratio, *Neu* neutrocyte, *NLR* neutrophil−lymphocyte ratio, *NOS* Newcastle–Ottawa Scale, *PLR* platelet-lymphocyte ratio, *Plt* platelet, *R* retrospective, *WBC* white blood cell

**Table 2:** Patient characteristics

Author	Sex (M; F)	Age	Follow up (month)	pT stage (≧3)	NAC	AC
Buisan	69; 9	NR	31	35 (46.7%)	75 (100%)	NR
Calvete	118; 3	68.1	51.4	80 (66.1%)	0	31 (25.6%)
Chipollini	776; 250	68.8	27.5	408 (39.8%)	387 (37.7%)	142 (13.8%)
D’Andrea	3362; 836	67	42.4	1853 (44.1%)	0	954 (22.7%)
Ergani	132; 16	65.7	21.12	70 (47.3%)	7 (4.7%)	NR
Gershman	1712; 374	68	132	678 (32.5%)	130 (6.2%)	192 (9.2%)
Gierth	551; 134	70	50	307 (44.9%)	0	NR
Gondo	158; 31	68.4	25.1	NR	0	NR
Gorgel	139; 14	61.65	NR	85 (50.4%)	NR	NR
Grimm	511; 153	70	24	NR	NR	NR
Ha	98; 20	69	34.1	NR	21 (17.8%)	NR
Hermanns	325; 99	70.1	58.4	194 (45.7%)	29 (6.8%)	87 (20.5%)
Jo	176; 24	67	28.6	NR	12 (6.0%)	NR
Jokisch	663; 203	70	38	410 (47.3%)	NR	NR
Kang	333; 52	66	NR	139 (36.1%)	0	96 (24.9%)
Kluth	747; 220	66	18	679 (70.2%)	0	279 (28.9%)
Ku	362; 57	65.1	37.7	177 (42.2%)	NR	NR
Kwon	636; 78	62.4	64.1	319 (44.7%)	0	164 (23.0%)
Lambert	153; 34	67.4	26.2	84 (44.9%)	35 (18.7%)	NR
Liu J	250; 46	61.71	72	102 (34.5%)	0	75 (25.3%)
Liu Z	164; 24	NR	38	69 (36.5%)	0	33 (17.5%)
Lucca	3240; 821	66.1	42	1912 (47.1%)	0	963 (23.7%)
Matsumoto	482; 112	67	48	251 (42.3%)	0	166 (27.9%)
Miyake	95; 22	72	22	43 (36.8%)	47 (40.2%)	20 (17.1%)
Moschini	754; 152	68	41	393 (43.4%)	0	NR
Ozcan	256; 30	60.7	28	124 (43.3%)	0	NR
Rajwa	115; 29	NR	14	NR	0	NR
Schubert	191; 55	NR	30	122 (49.6%)	0	40 (16.3%)
Sejima	214; 35	72	24.8	108 (43.4%)	0	16 (6.4%)
Tan	63; 21	67	30.1	43 (51.2%)	0	NR
Todenhofer	201; 57	NR	30	129 (50.0%)	0	41 (15.9%)
Un	254; 42	65.7	24.5	114 (38.5%)	0	NR
Viers	723; 176	69	130.8	347 (38.6%)	0	117 (13.0%)
Yang	275; 35	NR	71	NR	NR	242 (78.1%)
Yoshida	238; 64	70	81.6	134 (44.4%)	20 (6.6%)	62 (20.55)
Yuk	652; 119	64.8	84	255 (33.1%)	103 (13.4%)	173 (22.4%)

*AC* adjuvant chemotherapy, *F* female, *M* male, *NAC* neoadjuvant chemotherapy, *NR* not reported, *p* pathological

### Meta-analysis

#### Association of NLR with CSS in UCB

**Fig. 2:**
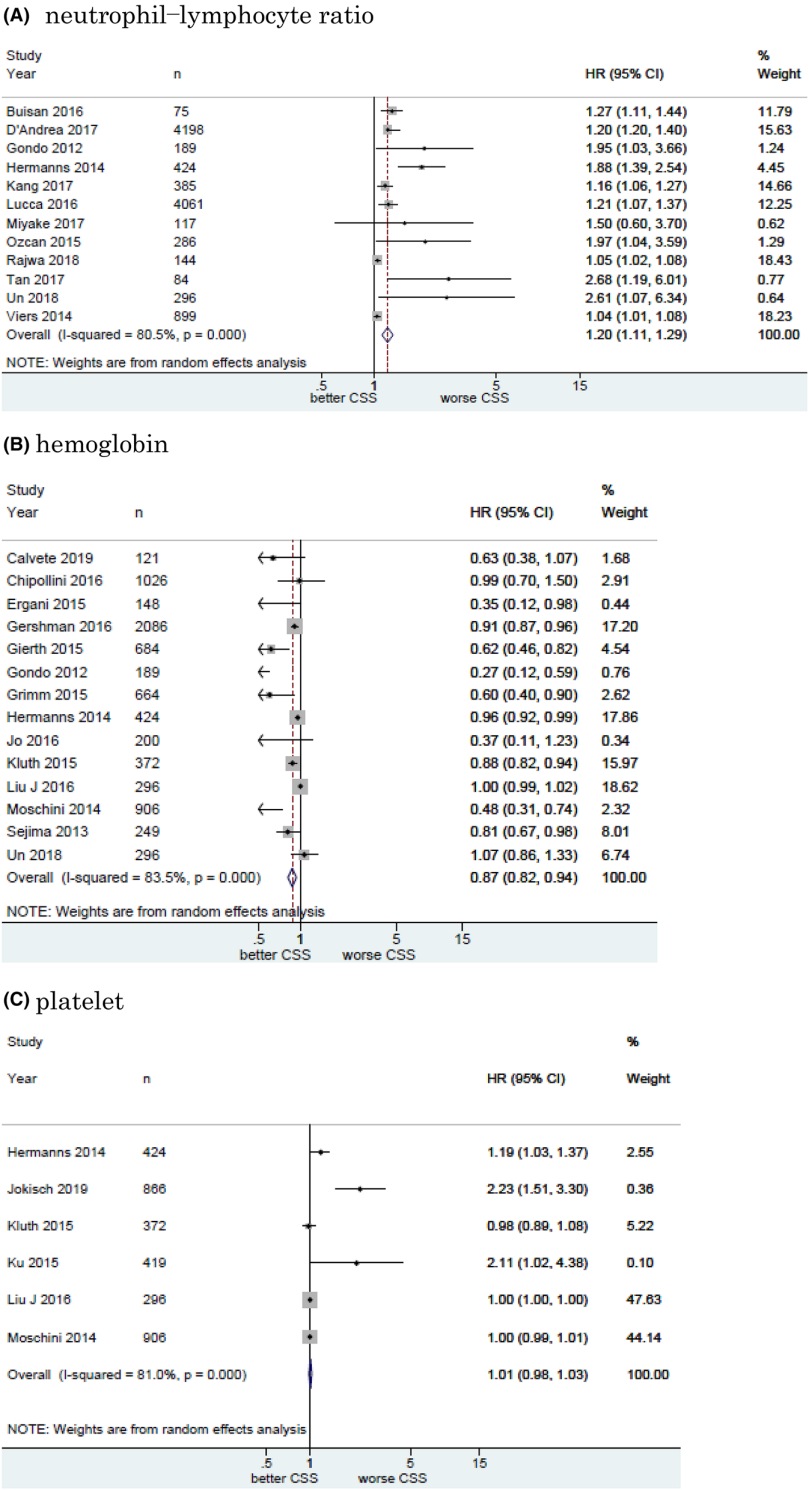

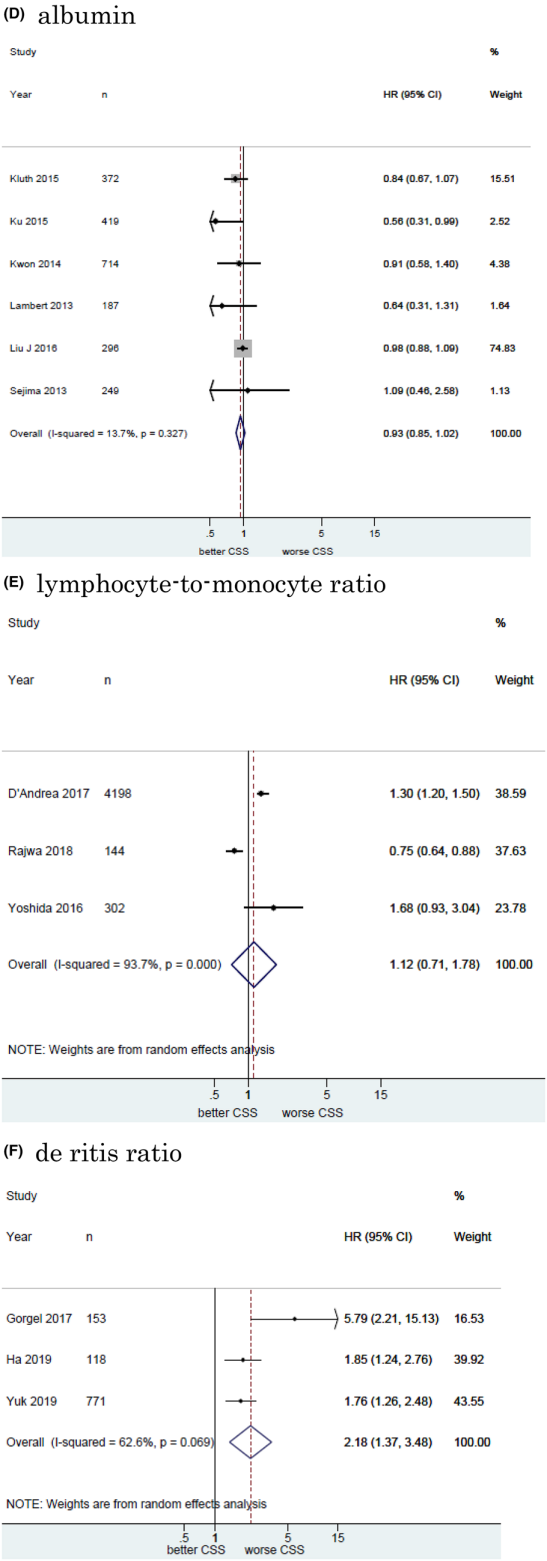

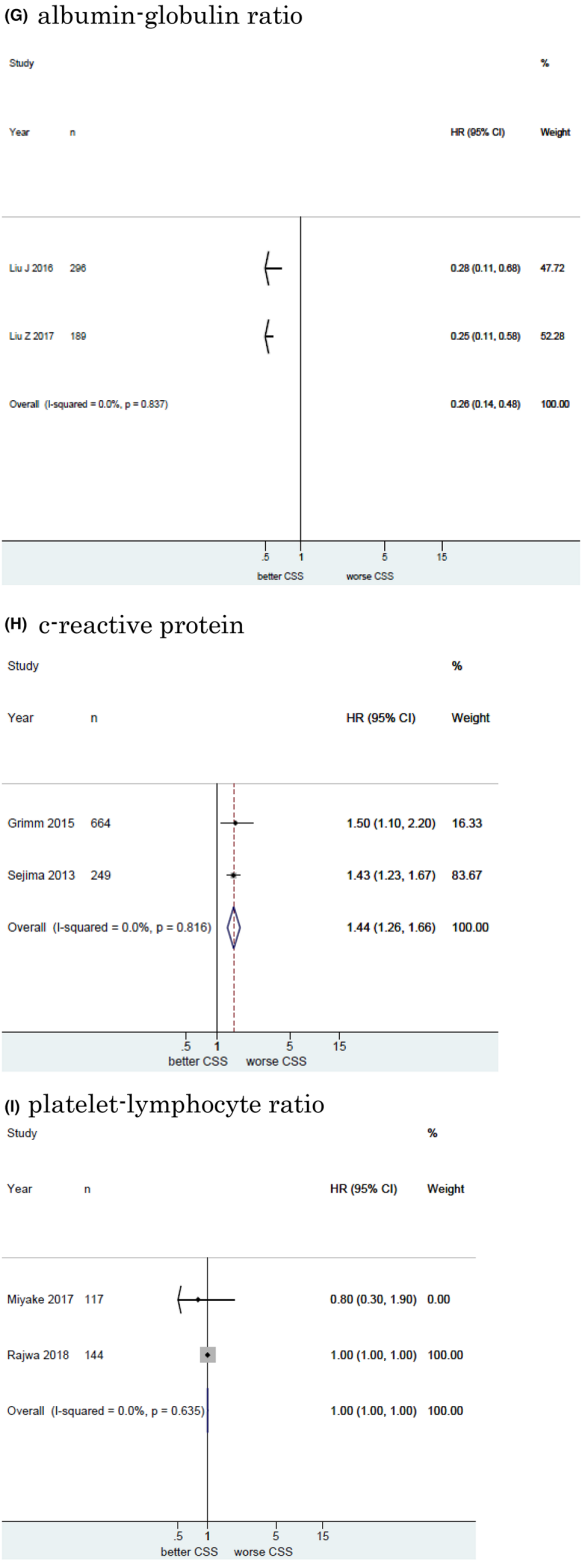

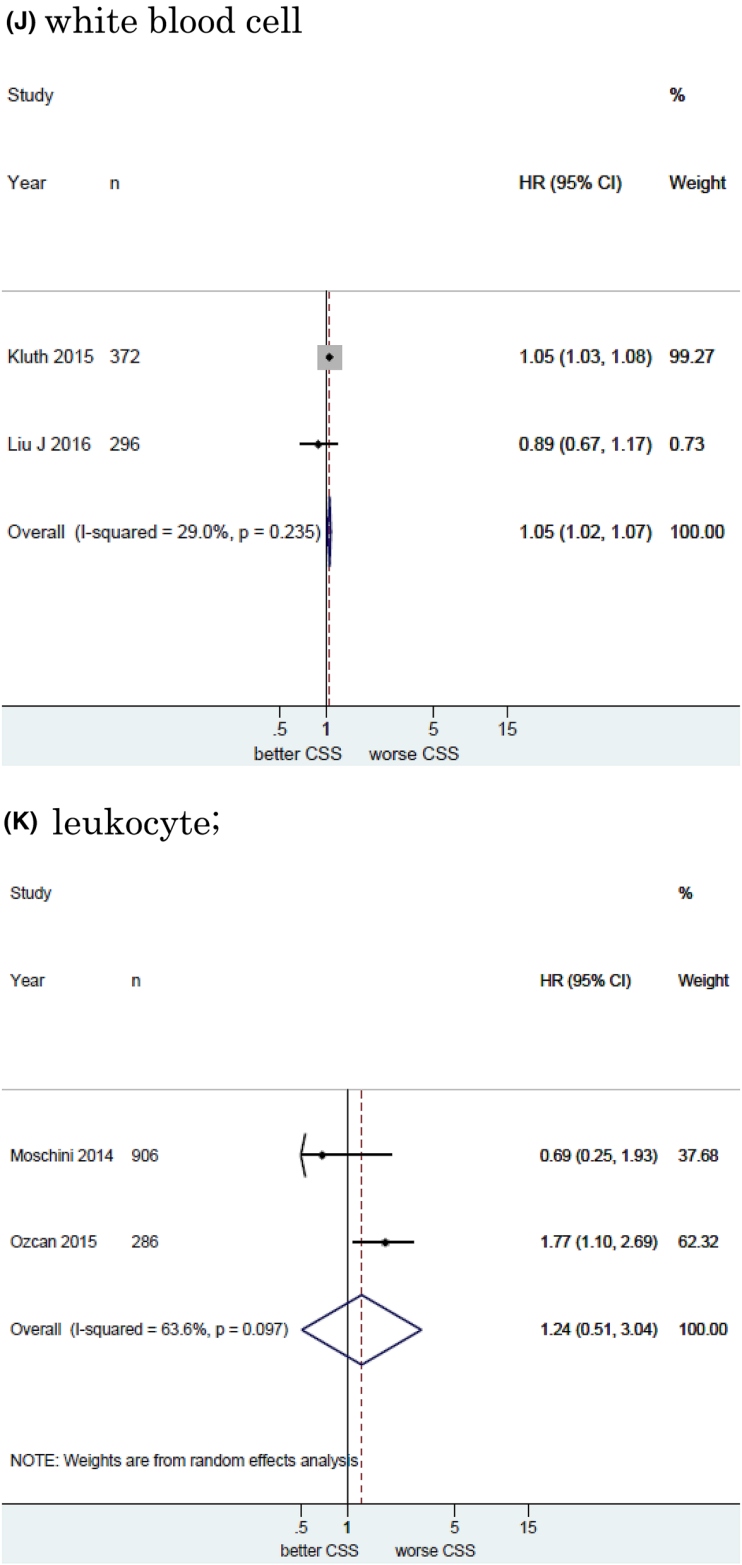
Forest plot (association of hematologic biomarkers with cancer-specific survival)**. a** neutrophil − lymphocyte ratio; **b** hemoglobin; **c** platelet; **d** albumin; **e** lymphocyte-to-monocyte ratio; **f** de ritis ratio; **g** albumin-globulin ratio; **h** c-reactive protein; **i** platelet-lymphocyte ratio; **j** white blood cell; **k** leukocyte

**Fig. 3:**
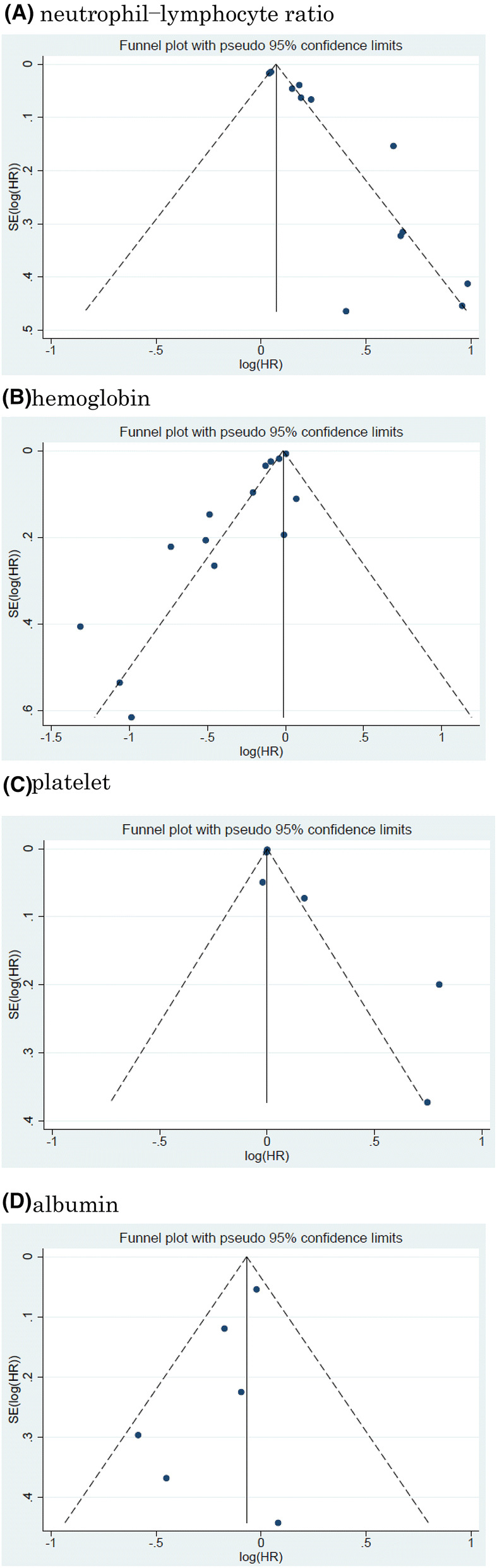

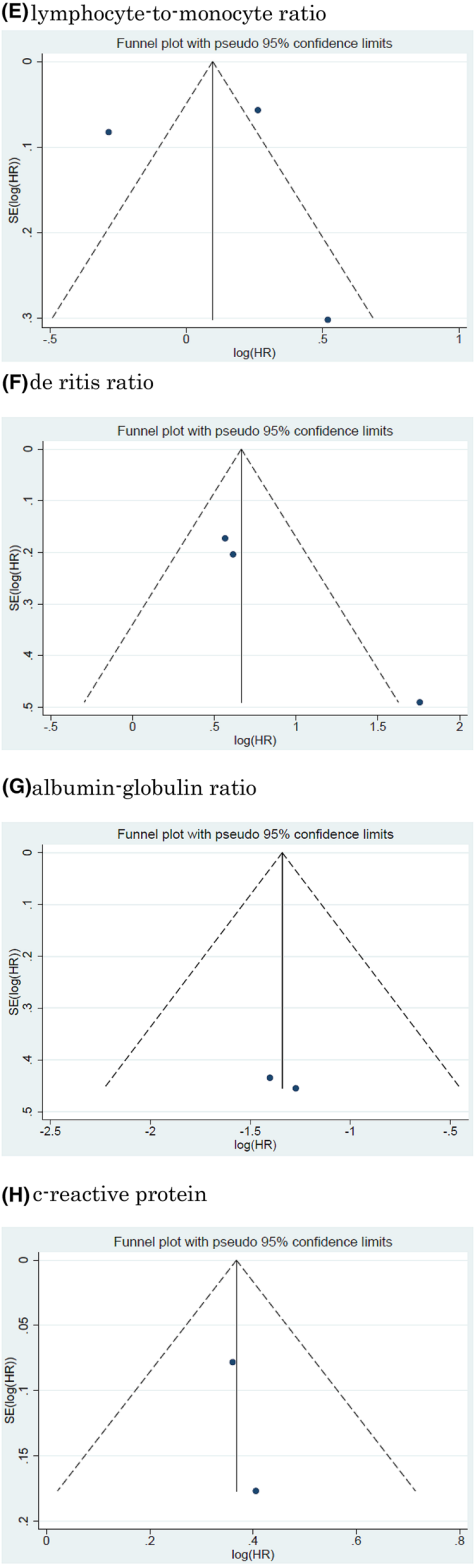

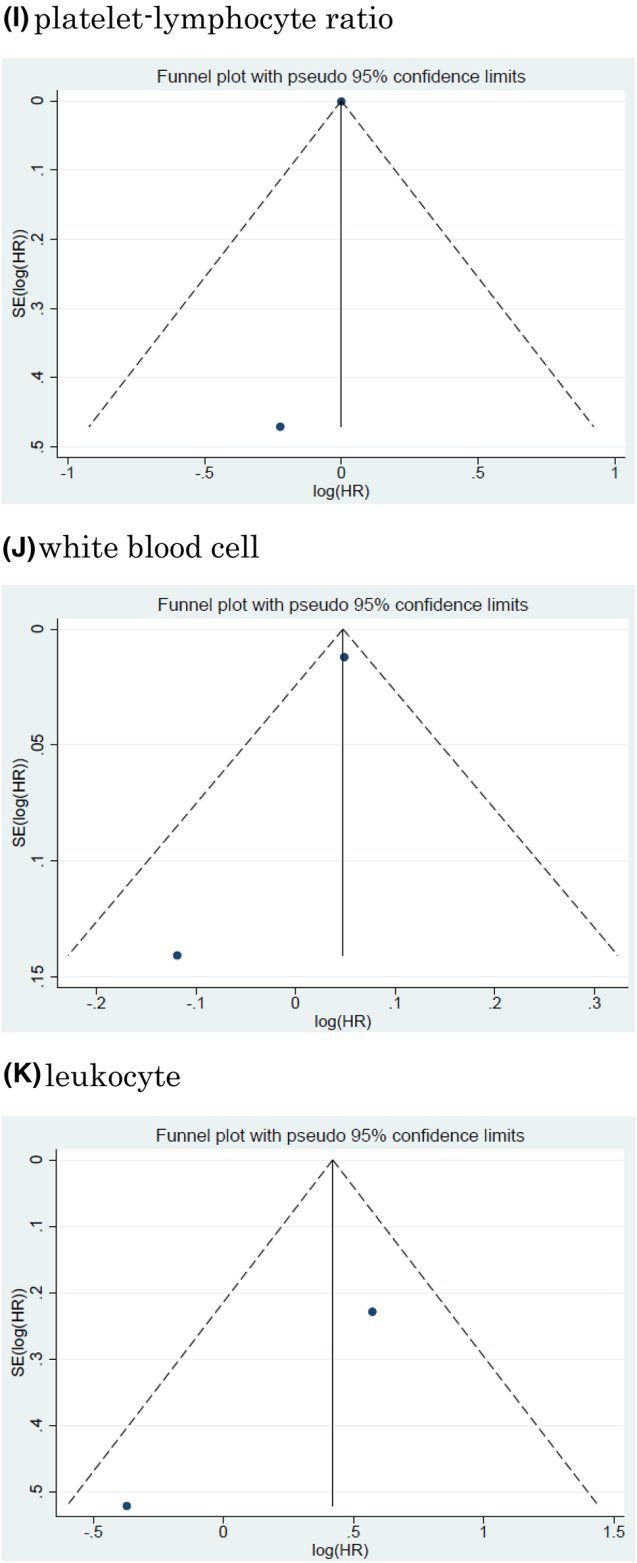
Funnel plot (association of hematologic biomarkers with cancer-specific survival). a neutrophil − lymphocyte ratio; **b** hemoglobin; **c** platelet; **d** albumin; **e** lymphocyte-to-monocyte ratio; **f** de ritis ratio; **g** albumin-globulin ratio; **h** c-reactive protein; **i** platelet-lymphocyte ratio; **j** white blood cell; **k** leukocyte

Twelve studies including 11, 158 patients provided data on the association of NLR with CSS in UCB. The forest plot (Fig. 2a) revealed that NLR was significantly associated with CSS in UCB (pooled HR: 1.20, 95% CI 1.11–1.29; *z* = 4.83). The Cochrane’s Q test (Chi^2^ = 56.41; *P* = 0.000) and *I*^2^ test (*I*^2^ = 80.5%) revealed significant heterogeneity. The funnel plot identified four studies over the pseudo-95% CI (Fig. 3a).

#### Association of Hb with CSS in UCB

Fourteen studies including 7661 patients provided data on the association of Hb with CSS in UCB. The forest plot (Fig. 2b) revealed that Hb was significantly associated with CSS in UCB (pooled HR, 0.87; 95% CI, 0.82–0.94; *z* = 3.71). The Cochrane’s *Q* test (Chi^2^ = 79.01; *P* = 0.000) and *I*^2^ test (*I*^2^ = 83.5%) revealed significant heterogeneity. The funnel plot identified six studies over the pseudo-95% CI (Fig. 3b).

#### Association of platelet count with CSS in UCB

Six studies including 3, 283 patients provided data on the association of platelet count (Plt) with CSS in UCB. The forest plot (Fig. 2c) revealed that Plt was not significantly associated with CSS in UCB (pooled HR: 1.01, 95% CI 0.98–1.03; *z* = 0.55). The Cochrane’s Q test (Chi^2^ = 26.31; *P* = 0.000) and *I*^2^ test (*I*^2^ = 81.0%) revealed significant heterogeneity. The funnel plot identified three studies over the pseudo-95% CI (Fig. 3c).

#### Association of albumin with CSS in UCB

Six studies including 2, 237 patients provided data on the association of albumin (Alb) with CSS in UCB. The forest plot (Fig. 2d) revealed that Alb was not significantly associated with CSS in UCB (pooled HR: 0.93, 95% CI 0.85–1.02; *z* = 1.45). The Cochrane’s *Q* test (Chi^2^ = 5.80; *P* = 0.327) and *I*^2^ test (*I*^2^ = 13.7%) revealed no significant heterogeneity. The funnel plot did not identify any studies over the pseudo-95% CI (Fig. 3d).

#### Association of LMR with CSS in UCB

Three studies including 4644 patients provided data on the association of LMR with CSS in UCB. The forest plot (Fig. 2e) revealed that LMR was not significantly associated with CSS in UCB (pooled HR, 1.12; 95% CI 0.71–1.78; z = 0.50). The Cochrane’s *Q* test (Chi^2^ = 31.73; *P* = 0.000) and *I*^2^ test (*I*^2^ = 93.7%) revealed significant heterogeneity. The funnel plot identified two studies over the pseudo-95% CI (Fig. 3e).

#### Association of De Ritis ratio with CSS in UCB

Three studies including 1042 patients provided data on the association of De Ritis ratio with CSS in UCB. The forest plot (Fig. 2f) revealed that De Ritis ratio was significantly associated with CSS in UCB (pooled HR, 2.18; 95% CI, 1.37 − 3.48; z = 3.30). The Cochrane’s *Q* test (Chi^2^ = 5.35; *P* = 0.069) and *I*^2^ test (*I*^2^ = 62.6%) revealed significant heterogeneity. The funnel plot identified one study over the pseudo-95% CI (Fig. 3f).

#### Association of Albumin-globulin ratio with CSS in UCB

Two studies including 485 patients provided data on the association of albumin-globulin ratio (AGR) with CSS in UCB. The forest plot (Fig. 2g) revealed that AGR was significantly associated with CSS in UCB (pooled HR: 0.26, 95% CI 0.14–0.48; *z* = 4.27). The Cochrane’s Q test (Chi^2^ = 0.04; *P* = 0.837) and *I*^2^ test (*I*^2^ = 0.0%) revealed no significant heterogeneity. The funnel plot did not identify any studies over the pseudo-95% CI (Fig. 3g).

#### Association of CRP with CSS in UCB

Two studies including 913 patients provided data on the association of CRP with CSS in UCB. The forest plot (Fig. 2h) revealed that CRP was significantly associated with CSS in UCB (pooled HR: 1.44, 95% CI 1.26–1.66; *z* = 5.15). The Cochrane’s *Q* test (Chi^2^ = 0.05;  *P* =  0.816) and *I*^2^ test (*I*^2^ = 0.0%) revealed no significant heterogeneity. The funnel plot did not identify any studies over the pseudo-95% CI (Fig. 3h).

#### Association of Platelet-lymphocyte ratio with CSS in UCB

Two studies including 261 patients provided data on the association of platelet-lymphocyte ratio (PLR) with CSS in UCB. The forest plot (Fig. 2I) revealed that PLR was not significantly associated with CSS in UCB (pooled HR: 1.00, 95% CI 1.00–1.00; *z* = 0.10). The Cochrane’s Q test (Chi^2^ = 0.22; *P* = 0.635) and I^2^ test (*I*^2^ = 0.0%) revealed no significant heterogeneity. The funnel plot did not identify any studies over the pseudo-95% CI (Fig.3I).

#### Association of White blood cell with CSS in UBC

Two studies including 668 patients provided data on the association of white blood cell (WBC) with CSS in UCB. The forest plot (Fig. 2j) revealed that WBC was significantly associated with CSS in UCB (pooled HR: 1.05, 95% CI 1.02–1.07; *z* = 3.95). The Cochrane’s *Q* test (Chi^2^ = 1.41; *P* = 0.235) and *I*^2^ test (*I*^2^ = 29.0%) revealed significant heterogeneity. The funnel plot did not identify any studies over the pseudo-95% CI (Fig. 3j).

#### Association of leukocyte with CSS in UCB

Two studies including 1, 192 patients provided data on the association of leukocyte with CSS in UCB. The forest plot (Fig. 2k) revealed that leukocyte was not significantly associated with CSS in UCB (pooled HR: 1.24, 95% CI 0.51 − 3.04; z = 0.02). The Cochrane’s Q test (Chi^2^ = 3.02; P = 0.097) and I^2^ test (I^2^ = 63.6%) revealed significant heterogeneity. The funnel plot did not identify any studies over the pseudo-95% CI (Fig. 3k).

### Other factors associated with CSS (in one paper only)

Estimate glomerular filtration rate (eGFR), and lymphocyte were significantly associated with CSS in one study each. Lactate dehydrogenase (LDH), and neutrocyte were found not to be significantly associated with CSS in one study each.

## Discussion

This systematic review and meta-analysis were conducted to investigate the prognostic value of preoperative hematologic biomarkers in UCB, based on their association with CSS. Study results indicate that high preoperative NLR, CRP, WBC, and De Ritis ratio, as well as low AGR, and Hb are significantly associated with worse CSS.

First, De Ritis ratio was found to be associated with CSS in UCB, potentially as a marker of cellular metabolism and cancer cell turnover. It is generally assumed that alanine aminotransferase (ALT) is more liver-specific, whereas aspartate aminotransferase (AST) is widely expressed in different tissue types [54]. Therefore, pathological conditions associated with tumor proliferation, tumor cell turnover, and tissue damage, are thought to be more likely to increase AST than ALT, thus making the AST/ALT ratio an attractive potential biomarker [55]. However, the exact mechanism underlying the correlation between elevated AST/ALT and poor prognosis in UCB patients remains to be elucidated. Most cancer cells rely on anaerobic glycolysis to generate the energy required for survival, growth and metastasis even in the presence of oxygen via a process referred to as the “Warburg effect” [56]. Furthermore, increased glycolysis has been shown to be linked to several alterations in mitochondrial activity involving NADH-related enzymes and glucose transporters, and high LDH and cytosolic NADH/NAD + have been shown to be essential for the maintenance of this enhanced glycolysis [57, 58]. AST is known to form part of the malate-aspartate shuttle pathway facilitating NADH/NAD + conversion [59]. Therefore, AST/ALT may be related to tumor metabolism in many glucose-utilizing malignancies, such as UC [60–62].

Second, AGR was found to be associated with CSS in UCB. Of the 2 major human serum proteins evaluated in AGR, albumin and globulin, albumin is generally used to assess nutritional status and severity of disease. Low albumin has been shown to reflect malnutrition, which is common among patients with cancer, leading to disruption of a number of human defense mechanisms, such as anatomic barriers, cellular and humoral immunity, and phagocyte function [63, 64]. Moreover, albumin is now considered a marker of inflammatory response in addition to a nutritional marker [65, 66]. Globulin (derived from total protein minus the albumin fraction) consists of various pro-inflammatory proteins, including CRP, complement components, and immunoglobulins, and is, therefore, a central component of immunity and inflammation. Chronic inflammation markers play an important role in the proliferation, progression, development, and metastasis of tumor cells. Thus, AGR, as a combination of 2 separate predictors of adverse outcome, may have greater predictive value, given that nutritional status and systemic inflammatory response are both implicated in the outcome of patients with UCB undergoing RC.

Third, as an index of hypoxia, Hb was found to be associated with CSS in UCB. Hypoxia, which is commonly seen in advanced tumors, represents an imbalance between oxygen supply and consumption and thus may contribute to the resistance of tumor cells to therapy, whose impact may also be further enhanced by anemia [67, 68]. Tumor hypoxia has been shown to induce expression of hypoxia-inducible factor 1α (HIF1α), which is known to be integral to adaptively responding to hypoxia by targeting many genes involved in facilitating tumor survival, proliferation, invasion, and metastasis [69–71]. Furthermore, research suggests that hypoxia may promote tumor progression by inducing genetic changes and clonal selection in tumor cells [72].

Finally, in addition to AGR, several markers of the systemic inflammatory response, such as CRP, WBC, and NLR were shown to be significantly associated with CSS in UCB. These markers are known to be stimulated by cancer-related inflammatory factors, such as interleukin-6 thus sensitively reflecting cancer-related inflammation [7, 73, 74]. Cancer and inflammation are linked through both extrinsic and intrinsic pathways, with the former being activated by infection or chronic inflammation, and the latter being driven by genetic changes, such as oncogene activation or tumor suppressor gene deactivation. Both pathways activate key transcription factors, primarily nuclear factor -kB, signal transducer and activator of transcription 3, and HIF1α in tumor cells, which in turn lead to inflammatory mediators and cyclooxygenase-2 being produced, resulting in cancer-related inflammation and further promotion of tumor progression [7]. Therefore, the elevation of these systemic inflammatory response biomarkers impacts cancer growth and development [75]. Moreover, not only above mentioned systemic inflammatory markers, anemia is also brought about by inflammation such as IL-6 [76]. Hypoxia due to anemia will lead to increased HIF1α, which then activate Glucose transporter 1 and Phosphofructokinase-2 involved in glycolysis, leading to an increase of De Ritis ratio [69, 77–79]. Thus, the hematological biomarkers we identified are all related to inflammation.

Although this meta-analysis revealed a strong association between several biomarkers and UCB mortality, it has some limitations that need to be taken into account. First, reporting bias could have led to non-publication of negative results. All the studies included were retrospective in design, thus increasing the risk of selection bias. Second, unknown pre-treatment factors (e.g., nutritional deficiencies, comorbidities, medications, and lifestyle factors) may have affected the hematologic biomarkers, thus producing systematic bias. Third, there were no established cut-off values for hematologic biomarkers among the studies evaluated, with the cut-off value being chosen by most investigators based on statistical methods (e.g., based on the highest sensitivity and specificity), the lower or higher limit of normal, or with pre-defined biomarker cut-off values from the literature. Fourth, the preoperative chemotherapeutic protocols were heterogeneous between the studies included, which did not allow each individual protocol to be assessed for its impact on the prognostic factors evaluated. In particular, it was a major limitation of the study that the hematologic biomarkers were not readily evaluable for their prognostic value in patients receiving and those not receiving NAC. Fifth, this systematic review and meta-analysis included no patients receiving immunotherapy. In this era of immunotherapy and other newly available targeted therapies, it remains unclear how the results of this meta-analysis may direct impact on patient management. Sixth, while it is crucial to examine hematologic biomarkers for their combined prognostic significance in UCB, this has not been adequately addressed in this systematic review and meta-analysis. It is a further limitation of the study that it was confined to the analysis of preoperative biomarkers, to the exclusion of relevant perioperative biomarkers. Seventh, despite its relevance, intravesical therapy prior to RC was not readily evaluable for its prognostic significance in UCB due to the paucity of data available from the literature. Finally, heterogeneity was detected in the CSS analysis, thus limiting the value of these results. Although the random effect model was used to address heterogeneity among the studies evaluated, the conclusions should be interpreted with caution. Therefore, well-designed prospective studies with long-term follow-up are required to validate the prognostic value of biomarkers in this setting, and to determine whether they could improve the current tools for risk stratification of patients with UCB.

## Conclusions

This meta-analysis revealed that several preoperative hematologic biomarkers were associated with an increased risk of cancer-specific mortality in patients with UCB. Therefore, it might be useful to incorporate such hematologic biomarkers into prognostic tools to help with appropriate risk stratification of patients with UCB. In addition, low AGR had the highest HR, suggesting indirectly potentially stronger prognostic value than any other biomarkers.
